# WHO's criteria for ethical health research priority-setting in the context of climate change

**DOI:** 10.2471/BLT.25.293973

**Published:** 2026-01-14

**Authors:** Bridget Pratt, Rieke van der Graaf, Gabrielle Samuel

**Affiliations:** a Queensland Bioethics Centre, Australian Catholic University, 1100 Nudgee Road, Banyo, Brisbane, Queensland 4014, Australia.; b Department of Bioethics and Health Humanities, University Medical Center Utrecht, Utrecht, Kingdom of the Netherlands.; c Department of Global Health & Social Medicine, King’s College London, London, England.

## Abstract

Health research on climate change has increased substantially in recognition of the impact of climate change on human health. This research raises new ethical questions for health research priority-setting, including how to prioritize research on climate change and health versus other types of health research unrelated to climate change, and how to prioritize among different health research projects focused on climate change. In this paper, we focus on the latter. We consider whether the ethical criteria for health research priority-setting recently proposed by the World Health Organization (WHO) should be used by funders when allocating resources among health research projects focused on climate change. The WHO criteria were developed in response to imbalances around who controls and sets health research agendas and who benefits from them. The four criteria for ethical research priority-setting are optimizing social value, following fair procedures, respecting special obligations and assessing risks. We first show that these criteria are relevant to priority-setting for research on climate change and health because evidence suggests that the above-mentioned imbalances may exist when allocating resources to climate change and health research. We next assess whether the four criteria can help reduce imbalances in who controls and who benefits from resource allocation to such research. Our analyses indicate that the WHO criteria can help if further specifications are included for research on climate change and health. We provide recommendations for how to further specify the criteria.

## Résumé

La recherche médicale liée au changement climatique s’est considérablement développée en raison de la prise de conscience de l’impact du changement climatique sur la santé humaine. Ce domaine de recherche soulève de nouvelles questions éthiques pour la définition des priorités en matière de recherche médicale, notamment quand il est question de hiérarchiser la recherche sur le changement climatique et la santé de manière distincte, par rapport à d’autres types de recherche médicale sans rapport avec le changement climatique, et de hiérarchiser différents projets de recherche médicale axés sur le changement climatique. Le présent article se concentre sur cette dernière question. Il examine la pertinence, pour les bailleurs de fonds, d’appliquer des critères éthiques pour la définition des priorités en matière de recherche sur la santé (critères qui ont été récemment proposés par l’Organisation mondiale de la Santé (OMS)) lors de l’allocation des ressources entre les projets de recherche médicale axés sur le changement climatique. Ces critères ont été élaborés en réaction aux déséquilibres entre les personnes qui définissent l’orientation des programmes de recherche médicale et celles qui en bénéficient. Les quatre critères permettant de définir les priorités en matière de recherche éthique sont l’optimisation de la valeur sociale, le respect de procédures équitables, le respect d’obligations particulières et l’évaluation des risques. Nous mettons tout d’abord en évidence la pertinence de ces critères pour définir les priorités en matière de recherche sur le changement climatique et la santé, car des éléments suggèrent que les déséquilibres susmentionnés peuvent survenir lors de la définition de priorités pour l’allocation des ressources à la recherche sur le changement climatique et la santé. Nous évaluons ensuite si les quatre critères peuvent contribuer à réduire les déséquilibres entre ceux qui allouent des ressources à la recherche sur le changement climatique et la santé et les bénéficiaires. Nos analyses indiquent que les critères de l’OMS peuvent être utiles si des définitions supplémentaires sont incluses pour la recherche sur le changement climatique et la santé. Enfin, nous formulons des recommandations sur la manière de définir davantage ces critères.

## Resumen

La investigación sanitaria sobre el cambio climático ha aumentado sustancialmente, en reconocimiento del impacto del cambio climático en la salud humana. Esta investigación plantea nuevas cuestiones éticas para el establecimiento de prioridades en investigación sanitaria, entre ellas cómo priorizar la investigación sobre el cambio climático y la salud frente a otros tipos de investigación sanitaria no relacionados con el cambio climático, y cómo establecer prioridades entre distintos proyectos de investigación sanitaria centrados en el cambio climático. En este artículo, se centra la atención en este último aspecto. Se analiza si los criterios éticos para el establecimiento de prioridades en investigación sanitaria propuestos recientemente por la Organización Mundial de la Salud (OMS) deberían ser utilizados por los financiadores al asignar recursos entre proyectos de investigación sanitaria centrados en el cambio climático. Los criterios de la OMS se elaboraron en respuesta a los desequilibrios en torno a quién controla y define las agendas de investigación sanitaria y quién se beneficia de ellas. Los cuatro criterios para el establecimiento ético de prioridades de investigación son la optimización del valor social, el seguimiento de procedimientos justos, el respeto de obligaciones especiales y la evaluación de riesgos. En primer lugar, se muestra que estos criterios son pertinentes para el establecimiento de prioridades en la investigación sobre el cambio climático y la salud, ya que la evidencia sugiere que los desequilibrios mencionados pueden existir al establecer prioridades para la asignación de recursos a la investigación sobre el cambio climático y la salud. A continuación, se evalúa si los cuatro criterios pueden contribuir a reducir los desequilibrios en cuanto a quién controla y quién se beneficia de la asignación de recursos a la investigación sobre el cambio climático y la salud. Los análisis indican que los criterios de la OMS pueden ser útiles si se incorporan especificaciones adicionales para la investigación sobre el cambio climático y la salud. Se formulan recomendaciones sobre cómo especificar con mayor detalle estos criterios.

## ملخص

شهدت الأبحاث الصحية المتعلقة بتغير المناخ زيادةً ملموسة نظرًا لتأثيره على صحة الإنسان. وتثير هذه الأبحاث تساؤلات أخلاقية جديدة حول ترتيب أولويات الأبحاث الصحية، بما في ذلك كيفية ترتيب أولويات الأبحاث المتعلقة بتغير المناخ والصحة مقارنةً بأنواع الأبحاث الصحية الأخرى غير المرتبطة بتغير المناخ، وكيفية ترتيب الأولويات بين مختلف مشاريع الأبحاث الصحية التي تركز على تغير المناخ. نحن نركز في هذه الورقة على تلك النقطة الأخيرة. ونبحث فيما إذا كان يجب على جهات التمويل استخدام المعايير الأخلاقية لترتيب أولويات الأبحاث الصحية التي اقترحتها منظمة الصحة العالمية مؤخرًا عند تخصيص الموارد بين مشاريع الأبحاث الصحية التي تركز على تغير المناخ. تم وضع معايير منظمة الصحة العالمية على سبيل الاستجابة لمواطن الخلل المتعلقة بمن يتحكم في أجندات الأبحاث الصحية ويضعها، ومن يستفيد منها. وتتمثل المعايير الأربعة لترتيب أولويات الأبحاث الأخلاقية في تحسين القيمة الاجتماعية، واتباع الإجراءات العادلة، واحترام الالتزامات الخاصة، وتقييم المخاطر. نحن نوضح أولًا أن هذه المعايير ذات صلة بترتيب أولويات البحث في مجال تغير المناخ والصحة، لأن الأدلة تشير إلى أن مواطن الخلل المذكورة بأعلى قد تكون موجودة عند ترتيب أولويات تخصيص الموارد لهذا النوع من الأبحاث. ثم نقوم بتقييم ما إذا كانت هذه المعايير الأربعة يمكنها أن تساعد في الحد من مواطن الخلل المتعلقة بمن يتحكم في تخصيص الموارد ومن يستفيد منها، ليقوم بأبحاث تغير المناخ والصحة. تُشير تحليلاتنا إلى أن معايير منظمة الصحة العالمية يمكنها أن تساعد في حال تضمين مواصفات إضافية لأبحاث تغير المناخ والصحة. نحن نقدم توصيات حول كيفية زيادة دقة هذه المعايير.

## 摘要

随着人们逐步意识到气候变化对人体健康的影响，针对气候变化开展的健康研究也随之大幅增加。我们在该项研究中就健康研究优先级设定提出了新的伦理问题，包括如何将气候变化和健康相关研究设定为级别高于其他与气候变化无关的健康研究的优先事项，以及如何设定专注于气候变化的不同健康研究项目的优先级。在本文中，我们重点讨论了后者。我们探讨了出资方在为专注于气候变化的各种健康研究项目分配资源时是否应采用世卫组织 (WHO) 近期提出的关于健康研究优先级设定的伦理标准。世卫组织制定此类标准的目的在于应对管控和制定健康研究议程的人员和对应受益人之间的权益不平衡现象。关于伦理研究优先级设定的四个标准分别是优化社会价值、遵循公平程序、履行特定义务以及评估各项风险。我们首先阐明，这些标准适用于气候变化和健康相关研究的优先级设定，因为相关证据表明，在为气候变化和健康研究设定优先级以分配资源的过程中，可能会存在上述不平衡现象。接下来，我们评估了这四个标准是否有助于减少管控分配至气候变化和健康相关研究的各种资源的人员和对应受益人之间的权益不平衡现象。我们的分析结果表明，如果世卫组织制定的此类标准中能对气候变化和健康相关研究进行进一步说明，则此类标准可能会颇有助益。我们针对如何对此类标准进行进一步说明提供了相关建议。

## Резюме

Исследования в сфере здравоохранения, связанные с изменениями климата, значительно расширились вследствие признания того влияния, которое климатические изменения оказывают на здоровье людей. Эти исследования поднимают новые этические вопросы, касающиеся приоритетности исследований связи климатических изменений со здоровьем человека по сравнению с остальными направлениями исследований в сфере здравоохранения (не связанными с изменениями климата). Рассматривается также приоритетность среди собственно тех исследований, которые относятся к теме изменения климата. Данная статья посвящена последнему вопросу. Авторы размышляют над тем, следует ли финансирующим лицам и организациям учитывать предложенные Всемирной организацией здравоохранения (ВОЗ) этические критерии приоритетности при распределении ресурсов на поддержку проектов, касающихся изменения климата. Критерии ВОЗ были разработаны как ответ на дисбаланс между тем, кто контролирует и формирует повестку дня в исследованиях здравоохранения, и тем, кому эти исследования приносят пользу. Четыре критерия этичной расстановки приоритетов в исследованиях таковы: оптимизация социальной пользы, соблюдение честных процедур, уважительное отношение к особым обязательствам и оценивание рисков. Сначала авторы показывают, что эти критерии релевантны в отношении определения приоритетности исследований связи климатических изменений со здоровьем, так как имеются доказательства наличия вышеупомянутого дисбаланса при выборе приоритетных направлений и распределении финансирования исследований в области климатических изменений и здоровья. Следующий шаг – оценка эффективности этих четырех критериев в сокращении дисбаланса между теми, кто контролирует выделение ресурсов на исследования связи климатических изменений со здоровьем,и теми, кому это приносит пользу. Анализ показывает, что критерии ВОЗ могут быть полезными, если в отношении исследований, посвященных связи климатических изменений со здоровьем, будут сделаны дополнительные уточнения. Предлагаются рекомендации таких дальнейших уточнений критериев.

## Introduction

Climate change is the greatest health threat of our time. As temperatures worldwide continue to rise, we are seeing more frequent heatwaves and bushfires, greater frequency and intensity of heavy precipitation events, increased risk of drought and reduced water availability, sea level rise, and further increases in ocean temperatures and acidification.^1^ Recognition of the impact on human health of these hazards has led to more research on climate change and its relationship to health. For example, in 2022, the United States National Institutes of Health provided an estimated 281 million United States dollars (US$) to research projects on climate change and health,^2^ and steps were taken in 2023 to increase the funding.^3^ While this funding is currently under threat due to the United States government’s recent approach to climate science,^4^ 15 963 studies on climate change and health are estimated to have been published globally between 2013 and 2019.^5^

Despite the increase in research on climate change and health, resources available for health research are generally limited, making choices about which research proposals to fund unavoidable. Health research priority-setting is a process through which decisions or recommendations are made about which health research questions or areas should be prioritized.^6^ In relation to research on climate change and health, priority-setting occurs between research on climate change and health and other types of health research unrelated to climate change. Priority-setting also occurs among health research projects on climate change. In this paper, we focus on priority-setting among research projects on climate change and health, and specifically, we focus on the allocation of resources at the level of research funding.

Within research on climate change and health, several types of studies are allocated resources. Health impact studies refer to research that aims to gain insights on how the hazards, exposures and impacts of climate change are affecting human health and health equity. Adaptation studies refer to research on adaptations to protect human health from the impacts of climate change. Mitigation studies refer to research on how health-related sectors can lessen their climate change impact and on the effect of mitigation (across all sectors) on health.

As affirmed by recent the World Health Organization (WHO) publication *Guidance on the ethics of health research priority-setting*^6^ and previously by ethicists,^7^^,^^8^ decisions about what health research priorities to set and how to allocate resources are a matter of ethical concern. As the guidance states,

*Health research has brought humanity tremendous benefits. But those benefits have not been evenly distributed. Enormous disparities remain built into the global research agenda. Control over what research is done remains in a small number of hands, often in high-income countries, with for-profit, non-profit and government-supported research all still disproportionately focused on conditions that affect populations that are better off… In the face of these concerns, there is an urgent need for those involved in health research to set priorities for that research in an ethical way.* ^6^

The WHO guidance was thus developed in response to imbalances around who controls and sets health research agendas, and who benefits from them. The guidance affirms an obligation to engage in ethically informed health research priority-setting that is grounded in considerations of justice.^6^ The guidance provides ethical criteria to guide the various actors who set health research priorities, including funders.^6^

In this paper, we consider the research question: should the ethical criteria for health research priority-setting recently proposed by WHO^6^ be used by funders when allocating resources to health research projects that are focused on climate change? To answer this question, we first show how WHO’s ethical criteria are relevant to priority-setting for research on climate change and health. We next assess whether applying those criteria can help reduce the trends of concern in research on climate change and health. Finally, we demonstrate that it is possible to apply WHO’s criteria if further specifications are included for research on climate change and health.

## Funding trends of ethical concern

Limited evidence exists on funding flows to research on climate change and health, and more broadly to climate change research. However, available data on resource allocation trends in these areas suggest the same patterns as in international health research and global health research generally,^9^^–^^15^ with control over the research and its benefits lying largely with high-income countries. In Box 1, we take evidence from the literature and offer our assessment of what trends of ethical concern are beginning to emerge in the allocation of funding to climate change and health research.

Box 1 **Trends of concern regarding funding of research on climate change and health**

Trend 1: funders mostly allocate resources to research on climate change and health in high-income countries, with comparatively little research done in the countries most vulnerable to climate change.  Evidence: two reviews of English-language research show this geographical distribution. From 2013 to 2019, 79.4% (12 629/15 914) of studies on climate change and health focused on high-income and upper-middle- income nations, such as China.^5^ A scoping review that included studies done from 2008 to 2019 found a similar pattern and concluded that communities where the impact of climate change is predicted to be most severe attract the least amount of research on climate change and health.^16^

Trend 2: the focus of most of the research on climate change and health may not align with the climate-related health needs and priorities of low- and middle-income countries.  Evidence: research on climate change and health in high-income countries shows an emphasis on chronic diseases, respiratory health and health-system demand.^5^ In contrast, studies done in low-income countries shows an emphasis on infectious diseases, food and nutrition, water, sanitation and hygiene, and maternal and child health.

Trend 3: funding may favour health impact studies over mitigation studies and adaptation studies. Evidence: a systematic review of climate–health research between 2013 and 2019 was dominated by impact studies (13380/15963 studies), with only a minority of studies focusing on mitigation (1660) or adaptation (1189).^5^ The same pattern was found in a scoping review of health research focused on climate change published between 2008 and 2019.^16^

Trend 4: funding may favour health research on high-technology solutions over low-technology or structural solutions.  Evidence: sustainability transitions are complicated by incentives that favour scientific or technological breakthroughs at the expense of low-technology (or nontechnological) solutions without prior systematic assessment of their social value.^17^ Additionally, the social determinants of the impact of climate change on health and modifiable entry points for intervention are underrepresented among the topics in the literature on climate change and health.^5^

Trend 5: funding from high-income countries is mostly allocated to climate and health research institutions based in high-income countries. Evidence: based on a sample of 1000 research projects granted funding between 1990 and 2020 of more than US$ 2.2 billion, funding for climate research projects has been largely allocated to the European Union (40.1%), United Kingdom of Great Britain and Northern Ireland (36.2%) and United States of America (9.8%).^18^ For research projects on climate change focused on countries included in the United Nations Economic Commission for Africa, , research institutions based in western Europe and the United States received 78.0% (US$ 480.25 million) of the funding from 1990 to 2020 compared with only 14.5% (US$ 89.15 million) for institutions based in the African Region. Thus, much of the funding for research on climate issues in the African Region originates outside Africa and goes to research institutions outside Africa.^19^ Five of the six countries that received the most funding for research on climate change over the period 1990–2020 were former British colonies.^19^

US$: United States dollars

Some of those trends, namely, resource allocation primarily to recipients in high-income countries or to research promoting technology-based solutions, may reinforce unfair power dynamics of subordination and colonialism,^20^^,^^21^ that is, control of decision-making and of political, economic and cultural systems.^22^^,^^23^ Even if researchers in high-income countries focus on matters of relevance to low- and middle-income countries, they may still decide what health research questions or topics should be prioritized in research projects. In other words, researchers from high-income countries control the agenda. Furthermore, where funding favours high-technology research, it reinforces economic and cultural models of high-income countries, namely, knowledge-based economic models that support increased investment in science and technology^24^ and product-driven solutions to health problems.^25^

Where resource allocation favours studies on health impact, less knowledge on effective mitigation and adaptation options is generated. Such knowledge is urgently needed for the benefit of populations in low- and middle-income countries because they are most affected by climate change. The lack of knowledge may hinder the design of evidence-based pathways to mitigate the health impacts of climate change^5^ and thereby reinforce distributive injustices, that is, the unequal risk of climate change affecting people’s health in low- and middle-income countries versus high-income countries.

Resource allocation to health research conducted in and relevant to high-income countries is another trend, and can further generate or worsen distributive injustices by ensuring that people in high-income countries disproportionately benefit from the results of health research projects focused on climate change. When research on climate change and health is largely conducted in high-income countries on topics of local relevance, this research is less likely to generate knowledge that will benefit populations in low- and middle-income countries. As a result, global disparities in the health impacts of climate change may widen (Box 1).

## WHO criteria

WHO’s ethical criteria for health research priority-setting are intended to help address imbalances in who controls and who benefits from health research. Therefore, we consider whether these criteria can reduce any of the trends of concern in Box 1. The WHO guidance proposes four ethical principles, or criteria, to underpin research priorities, including at the funding level: (i) optimize social value; (ii) follow fair procedures; (iii) respect special obligations; and (iv) assess risks^6^ (details available in online data repository).^26^ The environment is considered by two criteria: optimize social value and assess risks.^26^

### Social value

According to the WHO guidance, social value has three components: (i) the likelihood that the research will produce knowledge that will ultimately benefit human health and well-being; (ii) the magnitude of the benefits; and (iii) the extent to which providing the benefits will reduce inequities. The last two components are most relevant when allocating funding to research on climate change and health.

#### Magnitude of benefit

Assessing and comparing the magnitude of the benefits of health research requires knowing which benefits and beneficiaries should count, and the extent to which they should count relative to one another. Research ethics scholars have considered what benefits should count in terms of health and/or well-being. They have explored whether both or only health benefits should count, and whether direct and indirect health benefits or only direct health benefits should count. Scholars have also considered their relative value (that is, health benefits versus other well-being benefits, and direct health benefits versus indirect non-health benefits).^27^ Beneficiaries who count generally include people now and in the near future. However, research on climate change and health raises new questions about what benefits (that is, environmental benefits) and beneficiaries (that is, people in the distant future) should count and their relative value (that is, also including health benefits versus environmental benefits; direct health benefits versus indirect health benefits; present and near future beneficiaries versus distant future beneficiaries). So far, these questions have not been considered. They arise because mitigation studies generate environmental benefits and indirect human health benefits, including for people in the distant future, whereas health impact studies and adaptation studies generate direct human health impacts for people now and in the near future. Thus, the magnitude of benefits of research projects on climate change and health will vary depending on what benefits (that is, health, environmental, direct and indirect) and which beneficiaries (that is, people now, in the near future or in the distant future) should count and how much they should count.

The WHO guidance answers some of the new questions raised by research on climate change and health but not all. Indirect human health and well-being benefits and environmental benefits (possibly just to sentient non-human animals) should be considered, but the guidance does not specify how these benefits should be valued relative to direct human health benefits. Lack of guidance in this area is problematic because assignment of certain relative values can bias resource allocation in favour of health impact studies and adaptation studies over mitigation studies, or vice versa. If environmental benefits to ecosystems do not count at all, and/or environmental benefits and indirect human health benefits count much less, then assessments of magnitude of benefits will be biased in favour of research on climate change and health with direct human health benefits (that is, health impact and adaptation studies). An alternative, that the distant future is given the same weight as the present and near future, is also problematic because then mitigation studies will have a much greater number of beneficiaries than health impact and adaptation studies. In effect, the bias against either mitigation studies or adaptation studies seen in Box 1 would be reinforced. To reduce this bias, environmental benefits must be given substantial (but perhaps not equal) weight relative to health benefits, as must people in the distant future relative to people in the present and near future. Such a perspective on relative value is consistent with some non-anthropocentric positions and conceptions of environmental justice.^28^

#### Reducing inequities

The WHO guidance defines equity as, “the absence of unfair, avoidable, or remediable difference among groups of people” in relation to well-being (including health status) but notes that, “how equity is best specified will depend on the scope – geographical, topical and so on – of the priority-setting exercise.”^6^ For research on climate change and health, equity could be understood in terms of climate equity, that is, in terms of ensuring the absence of unfair, avoidable or remediable differences in risk of the impact of climate change on well-being among groups of people, including current, near future and distant future generations. Research on climate change and health would then promote climate equity by helping generate knowledge on how and why the different risks of the impact of climate change on health exist and how to eliminate them. The focus of such research would primarily need to be on the people most vulnerable to the negative health impacts of climate change. As such, understanding equity as climate equity would help reduce funding being allocated mostly to research projects on climate change and health conducted in high-income countries. This approach would also help increase resource allocation to mitigation and adaptation studies because mitigation and adaptation are both essential to reduce the unequal impact of climate change.^29^^,^^30^

Yet understanding climate inequity does not help counter trends against funding research on structural solutions or call for funding mitigation studies in high-income countries specifically. For these reasons, reducing injustice in general, and climate injustice in particular, is a better option as the third component of social value for research on climate change and health. As with climate equity, climate justice is understood in terms of well-being. Theories of justice in philosophy increasingly, but not always, define justice as ensuring a threshold level of well-being for individuals, groups and communities. Some priority is given to bringing disadvantaged groups up to that level of well-being.^31^^–^^33^ Addressing structural causes is especially important to alleviating disadvantage.^33^ Structural causes are unfair power relations, social norms, and social, economic and political institutions that create an unequal playing field where some people have to have to work much harder than others to achieve a good life.^22^

Climate justice then not only calls for reducing the unequal risk of climate change impacts on health and well-being,^29^^,^^30^ but also for giving some priority to tackling its structural or root causes.^34^^–^^36^ As with reducing climate inequity, reducing climate injustices would, therefore, give more value to projects in low- and middle-income countries, where vulnerability to climate change is higher.^29^ Reducing climate inequity would also prioritize research on climate change and health focused on how the structural causes of climate change affect human health. An example of such research would be studies on how current models of culture and development driven by fossil fuels harm our health.^37^

As a matter of climate justice, it has also been extensively argued that the burden of mitigation should not fall on the countries with the greatest health needs who are least responsible for climate change.^29^^,^^38^^,^^39^ Climate justice would then value mitigation studies in high-income countries higher than mitigation studies in low- and middle-income countries because they place the burden of mitigation on high-income countries. In contrast, understanding climate inequity could equally support increasing mitigation studies in high-income countries and low- and middle-income countries.

### Fair procedures

As we focus on resource allocation specifically, we have only considered the process of grant proposal evaluation by funders. The WHO guidance indicates that a fair process of grant proposal evaluation requires consideration of who should be panel members and in what numbers. From a climate change and health perspective, for epistemic reasons, people from low- and middle-income countries should be included on panels evaluating impact and adaptation studies. Equally, people from all countries should be included in panels evaluating mitigation studies. For intrinsic reasons, those populations most affected by climate change should be included in all panels, that is, people from low- and middle-income countries and future generations. Including future generations can take the form of thought models, procedural approaches or relational approaches.^40^^–^^43^

The WHO guidance also stipulates that meaningful inclusion occurs when participants, in this case, panel members in the evaluation process of grant proposals, are able to raise their voices and be heard. However, panel members’ level of participation, namely, who is part of the actual decision-making, is left open to funders to assign. In the context of research in climate change and health, we think the literature on procedural justice and climate change offers relevant guidance on this matter. In particular, it has been suggested that climate change decision-making processes should give vulnerable populations the power to shape adaptation decisions and the decision rules through which they are made. Granting this authority can also safeguard against exploitation by requiring conditions that ensure vulnerable populations are free from overt forms of domination or oppression.”^44^

Ensuring that representatives from low- and middle-income countries participate as decision-makers on grants’ panels and when selecting the rules for such panels could help counter certain trends in research on climate change and health that raise ethical concerns. This approach could help ensure resources are allocated to studies with a focus on low- and middle-income countries and their needs. This approach may also reduce the allocation of funds to researchers in high-income countries by diversifying which research projects are selected by the evaluation panel. At the same time, this approach will not address structural injustices that give applicants from high-income countries an advantage in preparing high-quality grant proposals. There is also a potential that a fair process, where decision-making is shared with representatives from low- and middle-income countries, could generate priorities inconsistent with the social value criterion and/or that reinforce trends in Box 1. Where ethical criteria conflict, the WHO guidance says trade-offs should always be made for justifiable reasons.^6^

### Special obligations

The WHO guidance states that special obligations are, “ethical duties that one party owes to another in virtue of their role, relationship, or history” but provides little additional detail on different types of special obligations.^6^ An analysis that was conducted on the special obligations of research funders is useful here.^45^ The authors categorize special obligations as constitutive or acquired duties. Constitutive duties derive from and align with a funder’s role in society. They exist for all types of funders. Acquired duties include duties of commitment, reciprocity and culpability.^45^ Duties of culpability are especially relevant in the context of climate change because they arise by virtue of past harmful interactions. In the following paragraphs, we discuss what constitutive and culpability duties arise for different types of funders with regard to priority-setting for research on climate change and health.

For governments, their constitutive duties are shaped by global justice,^6^^,^^45^ with governments having some obligations beyond their borders. Global justice thus supports government funders of high-income countries allocating resources to research on climate change and health in (and relevant to) all countries. Beyond constitutive duties, it can be argued that if a government has harmed a population, then it has a duty of culpability to compensate. This compensation can include conducting health research.^45^ Most governments of high-income countries would then have an acquired duty to fund research on climate change and health relevant to those populations most vulnerable to climate change because their countries are most responsible for climate change. If high-income countries fulfilled that duty, the trend we see favouring research in and relevant to high-income countries would be reduced.

Multilateral institutions, such as the Organisation for Economic Co-operation and Development, WHO and the World Trade Organization, also have constitutive duties to fund research on climate change and health in, and relevant to, low- and middle-income countries. These duties include funding research for populations whose research needs cannot or will not be met by states or other actors. In general, vulnerable populations will benefit the most from this research.^45^ If the mission of non-profit organizations is health-based and emphasizes the health of the people most in need worldwide, they arguably would have a constitutive duty to fund research on climate change and health in, and relevant to, low- and middle-income countries. The constitutive duties of private companies are not relevant to research on climate change and health because their duties are to their shareholders.

Duties of culpability may also be relevant to multilateral institutions, non-profit organizations and private companies, if they are considered to be complicit in incentivizing and perpetuating carbon-intensive economic activities driven by capitalism. Such complicity is arguably evidenced by private companies. A study estimated the aggregate global emissions of the pharmaceutical sector to be about 52 million tonnes.^46^ For that reason, pharmaceutical companies have an acquired duty to compensate populations most vulnerable to climate change and its effects, and hence to support research on climate change and health in, and relevant to, low- and middle-income countries.

### Assess risks

The WHO guidance requires consideration of whether health research projects pose a risk of third-party harms to humans, animals or the environment and whether those harms are justified.^6^ One aspect of particular relevance to research on climate change and health is that if the research leads to environmental (and consequently human health) harms, then low-technology intervention studies, such as providing free drinking water to avoid heat stress, may be preferred over high-technology intervention studies that use more resources. Applying this criterion could help reverse the ethically problematic trend of focusing on high-technology research. The WHO guidance states “new health technologies may have substantial and potentially deleterious effects if they are energy and resource intensive.”^6^

## Conclusions

Applying the WHO ethical criteria for health research priority-setting as we outline in Table 1 can help reduce certain trends of concern in allocation of funding for research on climate change and health. However, avoiding or further reducing these trends requires broader structural changes to the health research system and how grants programmes are designed. For example, to avoid allocation of disproportionately more funds to researchers in high-income countries for research on climate change and health, a fairer grant proposal evaluation processes is needed. Programmes need to be designed that are epistemically fair^47^ and have eligibility requirements or leadership requirements^48^ that favour applicants from low- and middle-income countries. Furthermore, structural injustices that give applicants from high-income studies an advantage in grant writing and management should be eliminated.

**Table 1:** Recommendations for further specification of WHO criteria for research on climate change and health

WHO criterion^6^	Proposed further specification for research on climate change and health	Funding trend in research on climate change and health reduced by the proposed specification
Social value (magnitude of benefits, likelihood of benefits and equity)	In assessing the magnitude of benefits and the relative value of benefits and beneficiaries, give environmental benefits similar (but perhaps not equal) weight relative to health benefits and give some (but not equal) weight to people in the distant future relative to people now and in the near future	Trend 3: resource allocation bias against mitigation studies and adaptation studies
	Rather than assessing the extent to which providing the benefits will reduce inequities, assess the extent to which providing the benefits will reduce climate injustice, that is, the unequal risk of climate change impacts on health and well-being	Trend 1: resource allocation to research done in high-income countries Trend 2: resource allocation to research relevant to high-income countries
	In assessing reduction of climate injustice, give some priority to studies that tackle structural or root causes of unequal risk of climate change impacts on health and well-being	Trend 4: resource allocation to research on high-technology solutions
Fair process (inclusion, transparency and accountability)	In achieving inclusion, representatives from low- and middle-income countries and future generations participate as decision-makers	Trend 1: resource allocation to research done in high-income countries Trend 2: resource allocation to research relevant to high-income countries Trend 5: resource allocation to researchers in high-income countries
Special obligations	In identifying special obligations, give specific consideration to constitutive duties and duties of culpability for governments of high-income countries, multilateral institutions, pharmaceutical companies and, where relevant, non-profit organizations	Trend 1: resource allocation to research done in high-income countries Trend 2: resource allocation to research relevant to high-income countries
Assess risks	No further specification is necessary	No further specification is necessary

WHO: World Health Organization.

(i) optimize social value; (ii) follow fair procedures; (iii) respect special obligations; and (iv) assess risks^6^
